# Supplementary material for: Extraction Methods Determine the Quality of Soil Microbiota Acquisition
Source: Microorganisms. 2024 Feb 17;12(2):403. doi: 10.3390/microorganisms12020403 (PMC10891820; doi:10.3390/microorganisms12020403)
Supplement: Supplementary file 1 [file microorganisms-12-00403-s001.zip › microorganisms-2850815-supplementary.pdf]

Table S1. CFU value of soil bacterial suspension extracted from CK、CF、UT1 and LT methods

treatment	CFU/ml
**CK**	**0.811±0.351a**
**CF**	**0.706±0.328a**
**UT1**	**1,939±0.516b**
**LT**	**0.744±0.294a**

Note: Based on one-way ANOVA with Tukey's test, the same letter means the difference is not significant， different letters indicate significant differences ($p < 0.0001$). The values are means ± SD. Eighteen repeats per extraction method. Groups are abbreviated as: control method, CK; Centrifugation method, CF; Sonication method, UT1; Oscillation method, LT.

Table S2. The number of culturable bacteria colony morphology species from CK, CF, UT1 and LT treatment

treatment	species
**CK**	**5.500±0.985a**
**CF**	**4.833±0.707a**
**UT1**	**8.333±0.907b**
**LT**	**5.333±0.840a**

Note: Based on one-way ANOVA with Tukey's test, the same letter means the difference is not significant， different letters indicate significant differences ($p < 0.0001$). The values are means ± SD. Eighteen repeats per extraction method. Abbreviations were same as **Table S1**.

Table S3. CFU value of soil bacterial suspension extracted from UT1、 UT2and UT3 methods.

treatment	CFU/ml
**UT1**	**1,939±0.516a**
**UT2**	**0.933±0.340b**
**UT3**	**1.978±0.542a**

Note: Based on one-way ANOVA with Tukey's test, the same letter means the difference is not significant， different letters indicate significant differences ($p < 0.0001$). The values are means ± SD. Eighteen repeats per extraction method. Groups are abbreviated as: Sonication method, UT1; Sonication method used new strategy, UT2; Increased sonication times of new strategy method, UT3.

Table S4. The number of culturable bacteria colony morphology species from UT1, UT2 and UT3 treatment

treatment	species
**UT1**	**8.333±0.907b**
**UT2**	**4.944±0.938a**
**UT3**	**7.722±1.018b**

Note: Based on one-way ANOVA with Tukey's test, the same letter means the difference is not significant， different letters indicate significant differences ($p < 0.0001$). The values are means ± SD. Eighteen repeats per extraction method. Abbreviations were same as **Table S2**.

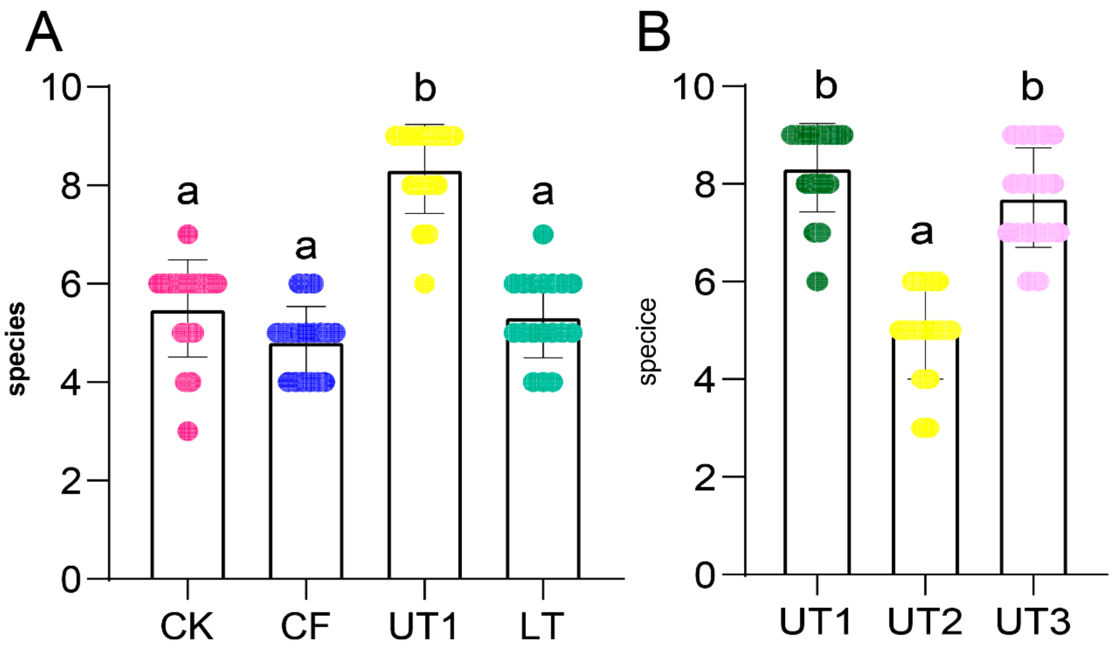

Figure S1. Extraction methods affect the number of culturable bacteria colony species. A. The number of culturable colony morphology species among CK, CF, UT1 and LT extracted microbiota. **B.** The number of culturable bacterial colony morphology species extracted through UT1, UT2 and UT3 methods. Letters indicate by one-way ANOVA and Tukey's HSD, the same letters indicated no significant difference, while different letters indicated significant difference ($p < 0.0001$). The scatter represents the sample data, bars represent mean ± SD. Eighteen repeats per extraction method. Groups are abbreviated as: control method, CK; Centrifugation method, CF; Sonication method, UT1; Oscillation method, LT; Sonication method used new strategy, UT2; Increased sonication times of new strategy method, UT3. Rose red, blue, yellow, green, dark green and pink indicated CK, CF, UT1, LT, UT2 and UT3, respectively.

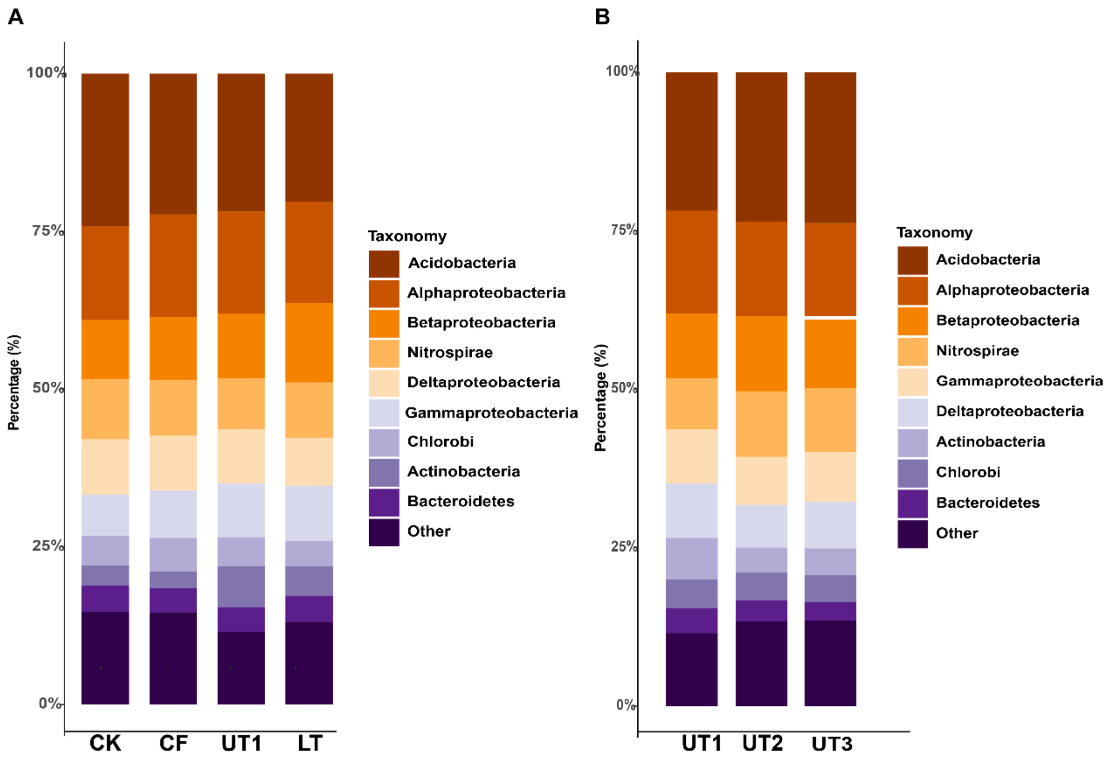

Figure S2. Relative abundance of bacterial communities at the phylum level. A. Relative abundance of bacterial communities extracted through CK, CF, UT1 and LT at the phylum level. **B.** Relative abundance of bacterial communities extracted through UT1, UT2 and UT3 at the phylum level. Each method repeated three times. Abbreviations were same as **Figure S1**.

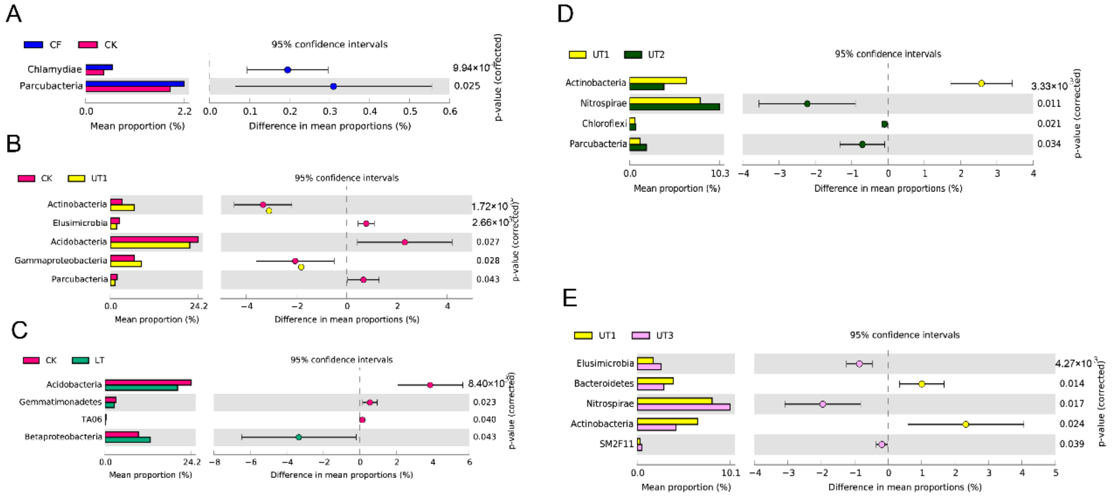

Figure S3. Group differences of bacterial species at phylum level between treatments. Differences analysis of top10 order among CK and CF extracted microbiota **(A),** CK and UT1 extracted microbiota **(B),** CK and LT extracted microbiota **(C),** UT1 and UT2 extracted microbiota **(D),** UT1 and UT3 extracted microbiota **(E).** The histogram showed the difference of species abundance between the two groups. The dot and bar plot shows the percentage of species between the two groups in each sample. The difference in proportions between groups is shown with 95% confidence intervals. Only $p < 0.05$ (Welch's t test), are shown and composition. Each method repeated three times. Abbreviations were same as **Figure S1**.

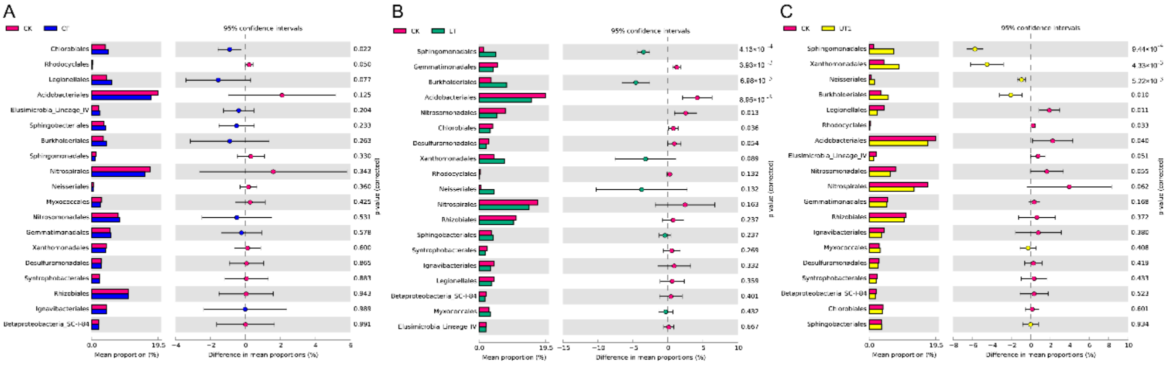

Figure S4. Group differences of bacterial species at the order level between different treatments. Differences analysis of top10 order among CK and CF extracted microbiota **(A),** CK and LT extracted microbiota **(B),** CK and UT1 extracted microbiota **(C).** The histogram showed the difference of species abundance between the two groups. The dot and bar plot shows the percentage of species between the two groups in each sample. The difference in proportions between groups is shown with 95% confidence intervals. Only p < 1 (Welch's t test), are shown and composition. Each method repeated three times. Abbreviations were same as **Figure S1.**

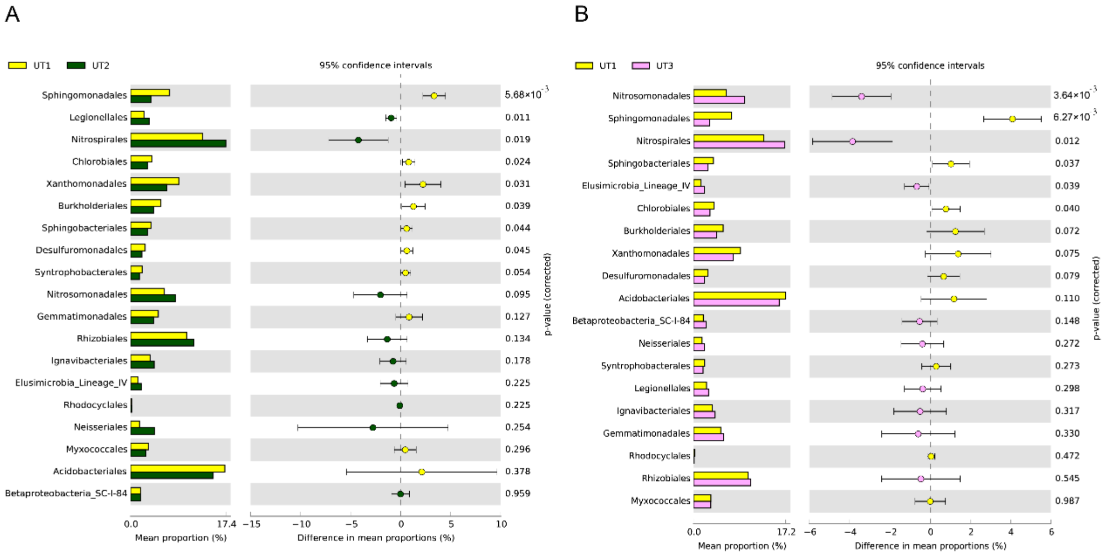

Figure S5. Group differences of bacterial species at the order level between different treatments. Differences analysis of top10 order among UT1 and UT2 extracted microbiota **(A),** UT1 and UT3 extracted microbiota **(B).** The histogram showed the difference of species abundance between the two groups. The dot and bar plot shows the percentage of species between the two groups in each sample. The difference in proportions between groups is shown with 95% confidence intervals. Only p < 1 (Welch's t test), are shown and composition. Each method repeated three times. Abbreviations were same as **Figure S1.**
